# Spectrum of Angle Closure, Uveal Effusion Syndrome, and Nanophthalmos

**DOI:** 10.5005/jp-journals-10008-1211

**Published:** 2016-10-29

**Authors:** Eric Areiter, Matthew Neale, Sandra M Johnson

**Affiliations:** 1 Resident, Department of Ophthalmology, Tulane Medical Center, New Orleans, Louisiana, USA; 2 Ophthalmologist, Department of Ophthalmology, University of Virginia School of Medicine, Charlottesville, Virginia, USA; 3 Resident, Department of Ophthalmology, University of Virginia School of Medicine, Charlottesville, Virginia, USA

**Keywords:** Angle closure glaucoma, Cataract, Glaucoma, Nanonophthalmos, Uveal effusion syndrome.

## Abstract

Nanophthalmos, uveal effusion syndrome, and acute angle closure glaucoma (ACG) can present as a continuum in a patient, as is described here. This patient's angle closure was thought to be caused by idiopathic uveal effusion syndrome, and while there are no generally accepted diagnosis criteria for nanophthalmos, our patient fulfilled the criteria as defined by Wu.^10^ To prevent development of further angle closure, the decision was made to do cataract extraction as opposed to medical management.

**How to cite this article:**

Areiter E, Neale M, Johnson SM. Spectrum of Angle Closure, Uveal Effusion Syndrome, and Nanophthalmos. J Curr Glaucoma Pract 2016;10(3):113-117.

## INTRODUCTION

Nanophthalmos is classically thought of as a rare condition characterized by a small eye in arrested development lacking many of the other malformations associated with eyes in the small-eye phenotype spectrum.^2-4^ The etiology of nanophthalmos is derived from developmental arrest of the globe, usually affecting both eyes, after the embryonic fissure is closed. This results in a variety of associated clinical features, including an abnormally thickened sclera, short axial length, small corneal diameter, and crowding of the anterior chamber secondary to a high lens to eye volume ratio.^5^ Angle closure glaucoma (ACG) is also associated with nanophthalmos, due to progressive shallowing of the anterior chamber and angle narrowing with relative pupillary blocking. This most commonly will develop when a nanophthalmic patient is in his 30s through 50s.^6^ When considering nanophthalmos, other diagnoses, such as simple and posterior microphthal-mos should also be examined. While nanophthalmos is associated with a small corneal diameter, posterior microphthalmos has a normal corneal diameter.^7^ Anterior microphthalmos has a horizontal corneal diameter of less than or equal to 11 mm.^8^

Nanophthalmic eyes are prone to problems related to their small biometry.^1,6,9^ No widely accepted biometric parameters for diagnosis exist; rather, the small-eye phenotype is best thought of as a clinical spectrum ranging from anophthalmos to axial hyperopia, and encompassing microphthalmos, nanophthalmos, segmental microph-thalmos, and blepharophimosis.^10^

Angle closure glaucoma is a group of disorders that share a common pathway of mechanical obstruction of the trabecular meshwork. Traditional risk factors for ACG include demographic, genetic, anatomic, and environmental factors.^11-14^ Demographic factors include increased age and female gender, both of which correspond with a shallower anterior chamber depth. Certain ethnicities, such as Inuits and East Asians (particularly Chinese from Singapore and Hong Kong) are predisposed.^14^ Hyperopia is also associated with a decrease in anterior chamber angle and ACG.

Static biometric dimensions may not fully account for anatomic risk.^5,15,16^ Certain anatomic parameters of the eye are in constant flux; specifically, the volume of the uveal tract is subject to change.^17^ Another slower but progressive biometric parameter is lens size. Commonly, this volumetric change refers to an expansion of the choroid, resulting in anterior rotation of the ciliary body. Dynamic changes in choroidal thickness can be caused by changes in posture, ocular surgery, and the valsalva maneuver.^18,19^ For nanophthalmic patients, management of ACG initially consists of laser iridotomy, which is performed to relieve pupillary block. Argon-laser peripheral iridoplasty can then be performed if the anterior angle continues to remain closed following laser iridotomy.^6^ Iridoplasty can however induce a significant inflammatory response, causing choroidal effusions in the nanophthalmic eye. Systemic steroids, cycloplegics, and miotics can also be used with caution.

Idiopathic uveal effusion syndrome is thought to result from an abnormality in the sclera that impedes transscleral intraocular fluid outflow and compresses the vortex vein, leading to congestion of the choroidal veins. Intraocular fluid then accumulates in the choroid, leading to ciliochoroidal detachment.^20^

Three subtypes of idiopathic uveal effusion syndrome have been described by Uyama. Type I consists of a nano-phthalmic eye with an average axial length of 16 mm. Type II is a non-nanophthalmic eye with abnormal sclera and average axial length of 21 mm and presenting with a small refractive error. Types I and II are both described by Uyama as having disorganization of collagen fiber bundles and deposits of proteoglycans in the matrix. Type III is a non-nanophthalmic eye with normal sclera lacking collagen disorganization or deposits.

Gass and Jallow introduced subscleral sclerectomy in 1983.^14^ Uyama showed that the technique is effective in Types I and II for inducing resolution of serous subret-inal fluid in these patients, while in type III this was not effective. Surgery on nanophthalmic eyes is infamous for complications and disappointment, however due to the lack of a generally accepted definition, patients should be evaluated individually for surgical eligibility. Ocular compression, choroidal effusions, pupillary block, and anterior chamber flattening must all be monitored for.

## CASE REPORT

A 46-year-old Caucasian male presented with acute redness, watering, and painful visual loss in the right eye oculus dexter (OD). The community ophthalmologist measured an intraocular pressure (IOP) of 60 mm Hg (Goldmann tonometer), and the patient was diagnosed with acute ACG. In addition to medical therapy, he was treated with laser peripheral iridotomy. Although IOP dropped into the normal range, the patient's anterior chamber remained very shallow. When corneal edema resolved, fundus examination revealed choroidal folds through the macula. He was referred to the Glaucoma Service at the University of Virginia with a diagnosis of nanophthalmos.

**Fig. 1:**
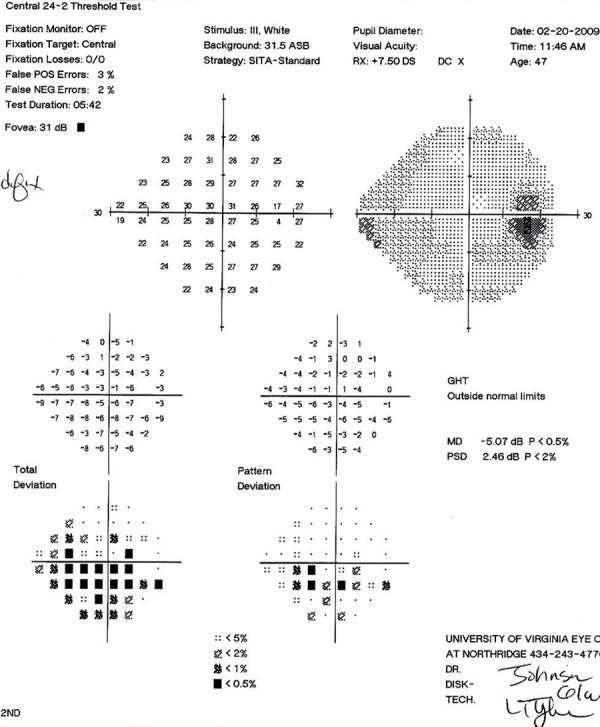
Automated static perimetr

Examination findings of our initial consultation are summarized in Table 1. Visual fields are shown in Figure 1.

The patient's angle closure was thought to be caused by idiopathic uveal effusion syndrome related to his short axial length and crowded anterior chamber. Although intraocular surgery in nanophthalmic eyes is relatively contraindicated, we felt that the risk of glaucoma recurrence was high enough to justify decompression of the anterior chamber by lens extraction. The patient received acetazolamide 500 mg by mouth preoperatively. Prior to entering the eye, a large scleral flap was fashioned in the nasal quadrant, and was left unsutured at the conclusion of the case. The sclera appeared to be of normal thickness; thus, a cut down to the uvea was not done.

**Fig. 2:**
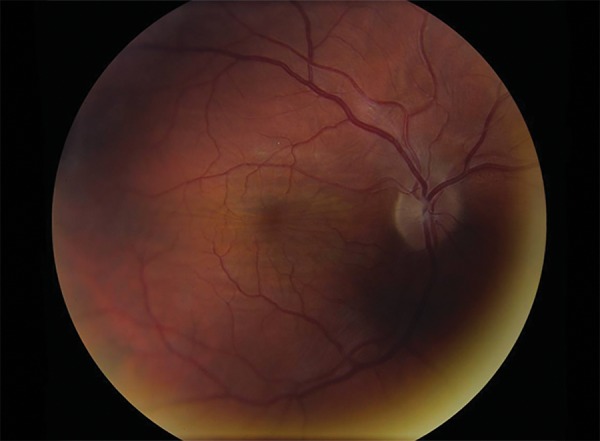
Fundus photograph of macular folds

Posterior synechiolysis, lensectomy, and a 34.0 D intraocular lens implantation were performed. Cycloplegia was maintained postoperatively. Postoperative day 1, folds were again noted in the macula. They persisted, and are demonstrated in the stereo fundus photograph seen in Figure 2.

**Table 1:** **Table 1:** Summary of ophthalmic examination

		*Oculus dexter*		*Oculus sinister*	
Va cc: (W: + 5.25 sph;		20/60		20/20	
+ 4.00 + 0.75 × 150)					
Manifest:		20/30 (+ 6.50 sph)		20/20 - 1	
				(+ 4 + 0.75 × 153)	
Tonometry		10 mm Hg		31 mm Hg	
Pachymetry		515 microns		533 microns	
Gonioscopy by		TM inferiorly only		TM nasally only	
Sussman					
Slit Lamp Biomicroscopy		Very shallow AC		Shallow AC	
		Corneal endothelial		LPI × 1	
		pigment			
		LPI × 2			
Fundus		Macular folds		Limited view	
		present			
Optic Nerve Cup to Disk		0.1		0.1	
Ratio					
Axial Length		19.58 mm		19.93 mm	
RNFL Thickness		130		128	
Anterior Chamber Depth		2.31		2.14	
Keratometry		46.87 and 48.35		46.87 and 48.15	
		@88, avg of 47.61		@90, avg 47.51	

**Fig. 3:**
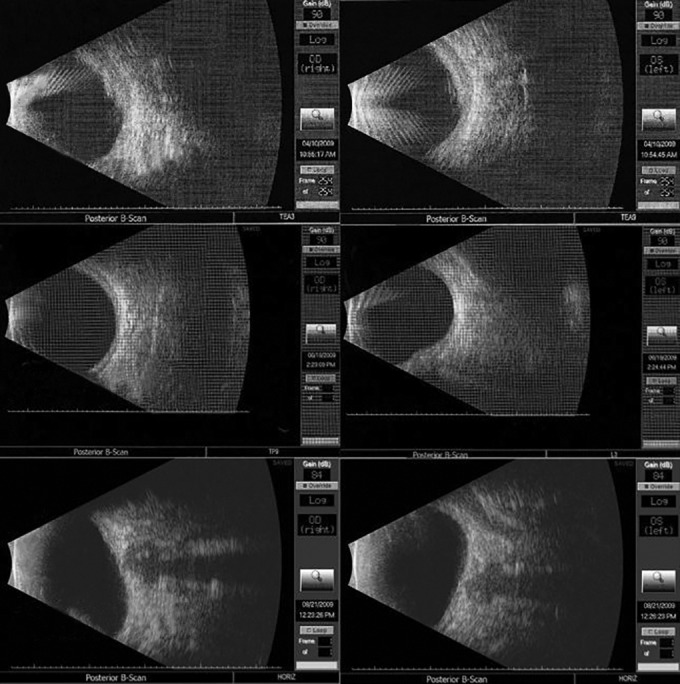
Serial echography showing gradual diminution in choroidal thickness

**Fig. 4:**
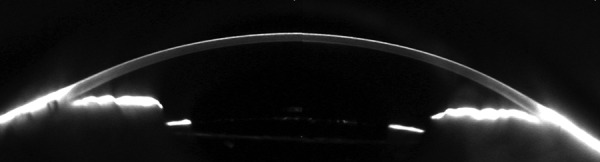
Postoperative Sheimpflug photography of the right eye

**Fig. 5:**
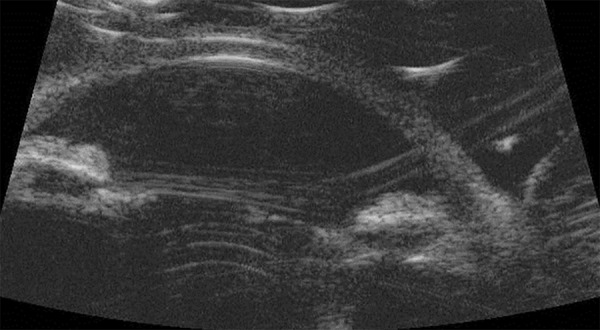
Anterior segment ultrasound showing anteriorly rotated ciliary processes

With retinal consultation, a diagnosis of idiopathic uveal effusion syndrome was confirmed. Echography is shown at 2, 4 months, and at 6 months postoperatively in Figure 3.

Although not obtained preoperatively, Scheimpflug photography and anterior segment ultrasound taken 7 months postoperatively show open angles and suggest postoperative deepening of the anterior chamber in Figure 4 and anteriorly rotated ciliary processes in Figure 5. Left eye was found to be stable for the past several years and patient was status quo postperipheral iridectomy for narrow angle as well as maintained on medical therapy.

## DISCUSSION

The presence of an expanded choroid in a small eye is most consistent with a diagnosis of idiopathic uveal effusion syndrome. Uyama had relatively strict criteria for the diagnosis of nanophthalmos. Eyes had to have axial length less than 19.0 mm, high hypermetropia, and thick, rigid sclera. The eye we report does not meet all of these criteria. Therefore, under Uyama's classification, this eye would be classified as type III (non-nanophthalmic eye with normal sclera). In Uyama's work, these eyes did not benefit from scleral surgery.

In other work, the definition of nanophthalmos has not been as firm. Wu considered eyes nanophthalmic if the anterior segment was crowded, if the eye was hyper-opic, axial length was less than 21 mm, and eye wall thickness in the posterior pole was greater than 1.4 mm. Our patient meets these criteria. Similarly, Yalvac et al^1^ diagnosed nanophthalmos based on an axial length of less than 20.5 mm, a shallow anterior chamber, high lens/eye volume ratio, moderate-to-severe hyperopia, and diffuse choroidal-scleral thickening as shown by echography.

The decision to perform cataract extraction was carefully made. Given that the primary defect was anatomic and not alleviated by laser iridotomy, and that an inferior arcuate scotoma was present, we decided that medical management was inadequate to prevent development of further angle closure. The more dense the lens and the more shallow the anterior chamber, the higher the risk of the corneal endothelium being jeopardized from the prior acute angle closure.^21^ Waiting for the development of elevated IOP would also contribute to the complexity of the cataract extraction. Laser gonioplasty would have been technically challenging due to unfavorable iris curvature. Phacoemulsification in nanophthalmic eyes is relatively safe, though patients should be thoroughly examined for complications due to their increased likelihood.^9,22^

**Table 2:** **Table 2:** Perioperative precautions for intraocular surgery on nanophthalmic eyes

*Preoperative*	
• Control of elevated blood pressure and choroidal inflammation to decrease the risk of postoperative choroidal effusion	
– Optimization of systemic blood pressure	
– Subtenons dexamethasone (1 mL of 10 mg/mL)	
– Oral prednisone 60-120 mg daily, depending on weight	
– Topical prednisolone acetate 1% every 2 hours while awake	
• If both cataract and glaucoma surgery are indicated, risk may be minimized by performing a combined procedure.	
• Intraocular lens (IOL) calculations should be done using SRK-T, Hoffer, and Holladay formulas, as they may be more accurate in highly hyperopic eyes.	
• Silicone IOLs may be superior to acrylic as they are thinner.	
*Intraoperative*	
• Intravenous mannitol at surgery to decrease vitreous pressure.	
• Ocular compression.	
• Creation of full-thickness anterior sclerotomies before entering the eye, and leaving them unsutured at the end of the case.	
• A thick viscoelastic should be used to maximally protect the corneal endothelium.	
• Phacoemulsification should be confined to the retropupillary and intrapupillary zones.	
• Creation of a large peripheral iridectomy to ward off pupillary block.	
• Goniosynechiolysis is controversial; one author cautions that the resulting increase in inflammation is not worth the potential benefit.	
• Viscoelastic should be left in the eye at the conclusion of the case to avoid hypotony in combined procedures.	
• Dexamethasone 10 mg subtenons, and methylprednisolone 250 mg IV at the conclusion of the case.	
*Postoperative*	
• Careful ophthalmoscopic exam for choroidal effusions; if present, admit the patient for	
– serial IOP and BP monitoring	
– high-dose IV steroids (methylprednisolone 250 mg IV every 6 hours)	
– maintenance of IOP in mid-20s (using intracameral viscoelastic injections to raise the IOP if necessary).	

While this eye was, by nature of its small biometry, predisposed to angle closure, it is possible that the accumulation of the choroidal effusion was the precipitating factor in the patient's acute presentation. As described by Quigley, this relatively small change in the volume of the uveal tract may cause an already crowded eye to cross the threshold into Acute angle closure glaucoma (AACG) by a volumetric displacement of the lens-iris diaphragm. Choroidal volume increases are accompanied with pressure increases within the corneoscleral shell and lens forward movement. Quigley also believed that many eyes share the feature of nanophthalmos of having choroidal expansion that leads to forward lens movement even if they are not small enough to be considered nanophthalmic.^19^

When faced with the dilemma of a nanophthalmic eye in need of intraocular surgery, extensive precautions have been proposed to reduce the risk of choroidal effusion and unstable IOP. While we did not implement more than a few of those listed, Table 2 is a consolidated list of interventions that may improve the surgical risk-benefit quotient. Although they may be helpful, note that these are expert recommendations, and not evidence based.^1,6,7,9^

## CONCLUSION

There seems to be a continuum between nanophthalmos, uveal effusion syndrome, and acute ACG as demonstrated by our patient. Overlapping nanophthalmos and uveal effusion syndrome in the absence of the effusions would present as a more straightforward primary angle closure patient.
